# Assessing Electronic Health Literacy in the State of Kuwait: Survey of Internet Users From an Arab State

**DOI:** 10.2196/11174

**Published:** 2019-05-24

**Authors:** Dari Alhuwail, Yousef Abdulsalam

**Affiliations:** 1 Department of Information Science College of Computing Sciences and Engineering Kuwait University Al-Adailiya Kuwait; 2 Health Informatics Unit Dasman Diabetes Institute Kuwait Kuwait; 3 Department of Quantitative Methods and Information Systems College of Business Administration Kuwait University Shuwaikh Kuwait

**Keywords:** eHEALS, literacy, health information, information-seeking, informatics, Arab, Kuwait

## Abstract

**Background:**

The internet and social media have become an important source for health information. In 2017, the State of Kuwait ranked first in mobile subscription penetration in the Arab world; nearly 90% of its population uses the internet. Electronic health (eHealth) literacy is important in populations that have easy and affordable access to internet resources to more effectively manage health conditions as well as improve general population health.

**Objective:**

The aim of this study was to assess eHealth literacy levels across internet users in Kuwait and identify demographic characteristics that influence eHealth literacy. Furthermore, the study aimed to identify the reasons and type of information that people seek online. Finally, this study examined the utilization of various social media channels for accessing online health information. The social media platforms considered were as follows: WhatsApp, Twitter, Instagram, YouTube, Facebook, and Snapchat.

**Methods:**

A cross-sectional anonymous Web-based survey was used to collect data about eHealth literacy and related information. The eHealth literacy scale (eHEALS), originally developed by Norman and Skinner, is measured using 8 Likert-type scales. A linear regression model estimates the effect of demographic variables such as age, gender, and education on eHealth literacy while controlling for participants’ perceived usefulness and importance of the internet. Participants were also surveyed about their frequency in using social media platforms for seeking health information.

**Results:**

Kuwait’s composite eHEALS, based on a sample of 386 participants, was 28.63, which is very similar to eHEALS observed among adult populations in other developed countries. Females in Kuwait demonstrated a higher average eHEALS compared with males. Among the social media platforms, the survey results indicated that YouTube is the most frequently used to seek health information, with Facebook being the least frequently used.

**Conclusions:**

Internet users in Kuwait appear confident in their ability to search for health-related information online compared with other populations, as indicated by aggregate eHEALS scores. Considering this finding, government and health care organizations should shift more efforts from traditional media toward online health information, focusing on the social media outlets that people in Kuwait find more useful for seeking health information.

## Introduction

Nearly 4.1 billion individuals have access to the internet worldwide [1]. Among its many purposes, the internet has become a popular destination for individuals seeking information about health, diet, and lifestyle [2-4]. Accordingly, there has been an uptick in the propagation of electronic health (eHealth) apps that support health care delivery [5], and the topic has received increased attention from health care institutions and health informatics researchers [6].

### Online Health Information as a Health Care Resource for Patients

Internet searches for health-related information online have been increasing exponentially, catalyzed by affordable access to the World Wide Web and the proliferation of mobile phones, including smartphones [7]. However, despite this large supply of health-related information online, many individuals are not able to use this information to make informed health decisions [8]. Seeking health information online requires eHealth literacy, which is defined as the ability to read, use computers, search for information, understand health information, and put it into context [9]. A prerequisite for one’s health management is an adequate level of knowledge about how to manage his or her diseases, conditions, and lifestyle[10-12]. Therefore, eHealth literacy becomes important for patients to be in charge of their health. Norman and Skinner [13] developed the eHealth Literacy Scale (eHEALS) to measure an individual’s skills in finding, evaluating, and applying online health information. This scale has since been widely adopted and tested in numerous contexts [14-23].

### Combating Noncommunicable Diseases With Information

Noncommunicable diseases (NCDs), such as diabetes and hypertension, have a great toll on health care systems [24,25]. Patients with NCDs must manage their conditions and adapt their lifestyles to gradually reduce the need for constant supervision of a health care professional [26]. Online health information is especially valuable for patients living with NCDs, who must eventually transition to self-management of their condition by seeking health information independently [27]. Providing patient-centered information resources to people with NCDs can save lives, improve quality of life, and ultimately reduce the burden on the health care system [28,29]. Therefore, it is essential that patients are able to independently identify, locate, process, and use the necessary information that can help them manage these diseases [14,30].

### Examining Electronic Health Literacy in an Internet-Savvy and Disease-Prone Population

The accessibility and widespread use of the internet and social media in the Middle East region can be a cost-effective mechanism for delivering health information to masses and creating support communities [31]. In this study, we consider the case of the State of Kuwait and the prevalence of eHealth literacy in Kuwait. This context is interesting because some of the population’s characteristics create great potential for eHealth literacy to augment traditional health care delivery channels. Kuwait is an oil-rich country with a high per capita gross domestic product and an affluent population that ranked first in mobile subscription penetration in the Arab world with nearly 90% of its population having access to the internet [32].

The World Health Organization reports that 72% of all deaths in Kuwait are attributed to NCDs [33]. The population in Kuwait struggles with numerous health issues such as high rates of obesity and other NCDs across most demographics such as diabetes (14.6% of the population), hypertension (15.7% of the population), and osteoarthritis (16%) [34]. Recent research reports that almost 40% of Kuwaiti citizens had prediabetes or diabetes [35].

The combination of internet, affluence, and high rate of NCDs provides a lucrative opportunity to empower patients and reduce health care system costs through the dissemination of online health information. For example, through the use of social media, patients, as well as their caregivers, can share personal information relevant to the disease, ask for help, and seek disease-specific emotional support [36,37].

This study targets the internet users in Kuwait and aims to (1) assess eHealth literacy rates, (2) gauge perceptions about the utility and importance of the internet as a source of health information, (3) determine demographic characteristics (such as age, internet usage, gender, and education) that influence eHealth literacy rates, (4) identify reasons for seeking health information online, and (5) examine the frequency of using social media channels to seek health information.

## Methods

### Study Design

This cross-sectional study [38] was initiated after obtaining the necessary ethical approvals from the Research Ethics Committee at Kuwait University, and it was conducted in full accordance with the World Medical Association Declaration of Helsinki. A convenience sampling approach was followed to recruit participants from the general population in Kuwait between September and October 2017 based on 3 inclusion criteria: respondents were aged 18 years or older, resided in Kuwait, and agreed to voluntarily participate in this anonymous study.

### Survey Instrument

The Web-based, self-administered, voluntary, and anonymous questionnaire consisted of the following 5 parts:

First, it involved collecting demographic information including age, gender, education, and internet use.

Second, respondents were asked about the reasons for seeking online health information and the types of health information sought online. Under each of these categories, participants provided binary, yes or no, answers to choices related to that category.

Third, the 8 items of the eHEALS were included [13]. Respondents rated their level of agreement with each of the 8 items on a scale of 1 to 5, with 1 being the lowest and 5 being the highest. Therefore, the theoretical range of the composite score is between 8 and 40. Furthermore, 2 supplemental eHEALS questions measuring perceived usefulness and importance of accessing health information online, used in similar studies [39], were also included. These questions were also measured on a scale of 1 to 5.

Fourth, following previous studies, 2 additional questions related to the reasons for and types of information sought online were included [15,40].

Finally, participants rated, on a scale of 1 to 5 (1=*Never*, 5=*Always*), their frequency in using social media platforms as a venue for seeking health information. Participants rated the following 6 social media platforms: WhatsApp, Twitter, YouTube, Snapchat, Instagram, and Facebook.

Bilingual faculty members in the Information Science and the Health Informatics disciplines translated the survey instrument from English to Arabic. Initially, the Information Science faculty member translated the survey from English to Arabic and then a Health Informatics faculty member checked this translation. A graduate student back-translated the survey from Arabic to English. The back-translated survey was checked for accuracy and clarity and approved by the researchers. Moreover, 10 individuals, who were colleagues of 1 of the researchers, were contacted to pilot the survey with the researcher face to face. In addition, 5 individuals tested the English version of the survey, and the other 5 individuals tested the translated Arabic version. Feedback from the pilot survey provided valuable insights to clarify concepts, questions, design, and the structure of the final survey instrument.

### Data Collection

The survey was published online using LimeSurvey hosted on the researchers’ server. The online survey was made available in both the Arabic and English languages.

A broad combination of strategies similar to those used by other studies was used to identify the convenience sample [41]. In addition to disseminating the survey to the researchers’ contacts and to capitalize on the heavy traffic around universities and shopping centers, open invitations to participate were circulated via printed posters at these locations. The posters were strategically displayed at the front entrance of 8 shopping centers distributed across Kuwait’s 6 governorates. The posters were also displayed on student boards across 11 colleges in Kuwait’s state university. The recruitment posters at universities allowed inviting a mixed group of students, faculty, and staff who were likely to use the internet given the nature of higher education. The recruitment posters at shopping centers allowed to expand the sampling pool to include a diverse set of the general population in Kuwait who regularly visit the cooperative store but may not currently attend a university or have graduated from a university.

The researchers also circulated electronic invitations through various mediums including emails, short message service (SMS) text messages, and social media. Initially, the researchers sent email invitations to their network of family, friends, and colleagues, inviting them to participate and help pass on the invitation to others. SMS text messages were sent via WhatsApp to an expanded network of the researchers’ contacts, especially as some potential participants may not use or check emails. To recruit participants via social media, the researchers mainly used Twitter to announce the study. The researchers approached major Twitter accounts followed by people in Kuwait to help voluntarily tweet or retweet about the study and how to participate.

### Data Analysis

The survey data were analyzed using the R software (version 3.5) developed by R Core Team [42]. The eHEALS score was analyzed, and its distribution was examined. Ordinary least squares regression was utilized to assess how demographics and perceptions can influence the eHEALS. Factors of interest were collected via the survey to examine their correlation with eHEALS. Factors included in the analysis were age, gender, education, and internet usage. We controlled for the perceived usefulness and perceived importance of the internet because these 2 measures were significant correlates to the eHEALS.

*P* values for all statistical tests were reported, and we considered an alpha of .05 when reporting that a test statistic is significant. In other words, we consider statistical tests to be significant when they show a *P* value of less than .05.

## Results

### Respondents' Characteristics

Participant demographic information is shown in Table 1. In total, 615 people attempted the survey, and 386 fully completed it (completion rate of 62.7%, 386/615). Of the respondents, 63% (243/386) were females, and 37% (143/386) were males. Furthermore, 63% of participants (244/386) accessed the internet for 3 hours or more per day. Compared with the general population in Kuwait, more females participated in the survey than males. In terms of age, our sample was more skewed toward older participants compared with the general population in Kuwait.

### Electronic Health Literacy

The eHealth literacy score, which was calculated as the composite of the 8 items, had a mean of 28.63, median of 29, and SD of 5.6. The dispersion of the observations appears normally distributed with a range between 8 and 40.

### Instrument Dimensionality

We ran a confirmatory factor analysis for a single factor model where all 8 items load onto 1-factor and a 3-factor model. The 3-factor model is based on recent studies that parse the eHEALS items into subgroups [43,44]. The fit statistics of the 2 models are presented in Table 2. We found that the 3-factor model demonstrated a better fit based on the global fit indices. However, the correlations between the 3 factors were statistically significant, indicating that they are subscales to an overarching unidimensional structure.

**Table 1:** Respondents' demographics (n=386).

Demographic		Statistics, n (%)	Kuwait population, %^a^
**Gender**			
	Male	143 (37.0)	61.2
	Female	243 (63.0)	38.7
**Age (years)**			
	0-19	22^b^ (5.7)	26.7
	20-29	113 (29.3)	10.7
	30-39	121 (31.3)	22.8
	40-49	56 (14.5)	22.4
	50-59	57 (14.8)	11.4
	60-69	16 (4.1)	4.4
	70-79	1 (0.3)	1.2
**Education level**			
	Primary school or lower	8 (2.1)	—^c^
	High school	69 (17.9)	—
	Diploma	43 (11.1)	—
	Bachelor’s degree	185 (47.9)	—
	Master's degree	48 (12.4)	—
	Doctorate degree	33 (8.5)	—
**Occupation**			
	Student	82 (21.2)	—
	Employed	232 (60.1)	—
	Unemployed	26 (6.7)	—
	Retired	46 (11.9)	—
**Internet use**			
	Less than 1 hour per day	32 (8.3)	—
	1-3 hours per day	110 (28.5)	—
	3-5 hours per day	99 (25.6)	—
	5+ hours per day	145 (37.6)	—

^a^Kuwait population statistics are from the Kuwait Central Bureau of Statistics estimate for January 1, 2018.

^b^Respondents in this group were either 18 or 19 years old.

^c^Data not available.

**Table 2:** Confirmatory factor analysis.

Goodness of fit statistics	1-Factor model	3-Factor model
Comparative fit index	.899	.931
Tucker-Lewis index	.858	.886
Root mean square error of approximation	.142	.128
Standardized root mean square residual	.053	.045

In response to the item *How useful do you feel the internet (including social media) is in helping you in making decisions about your health?,* 81% (312/386) of the participants considered the internet useful or very useful. In response to *How important is it for you to be able to access health resources on the internet (including social media)?,* 77% (296/386) of the participants expressed that it was important (or very important) to be able to access health resources on the internet.

The 2 items are correlated with one another with a correlation coefficient of .52. In addition, both perceived usefulness and perceived importance of the internet correlate highly with the eHealth literacy score, at .426 and .431, respectively.

### Factors Related to Electronic Health Literacy

Demographic characteristics were considered when investigating the study’s third aim of determining any factors that contribute to eHealth literacy rates. The results of the regression model are presented in Table 3. The model was statistically significant, explaining 28% of the variance in eHEALS observations (*F*_11,374_=12.29, *P*<.001).

*Age* (as measured by year of birth) was not a significant predictor of eHEALS after controlling for other factors. With regard to *gender*, the model predicts that males have a lower eHEALS score than females (β=−.23, *P*<.05) after controlling for other factors.

In terms of *education*, individuals with a Doctorate degree are expected to have a higher eHEALS score compared with bachelor’s degree holders by an average of 3.5 points, controlling for all other factors. Finally, individuals who used the internet more than 5 hours a day on average are expected to have a higher eHEALS compared with individuals who used the internet an average of 1 to 3 hours daily.

### Types and Reasons for Seeking Health Information Online

In addition to measuring the eHEALS score, the study aimed to identify the type of health information sought via the internet and social media, as well as the reasons that the participants sought them. The top health information sought online by participants included information about a *disease or medical problem* and *medical treatment or procedure*. The least common types were *online support group* and *search for a particular physician or hospital* (refer to Table 4).

In terms of reasons for seeking health information online, most participants cited reasons related to having a general curiosity about a topic (ie, *to be more informed* and *just out of interest*). On the other hand, the least common reasons for seeking health information online were *limited time with health professional* and *disagree with health professional's opinion* (refer to Table 5).

**Table 3:** Regression model predicting electronic health literacy (n=386).

Variable^a^		B (SE)	*β*	*t* (df)	*P* value
Intercept		25.93 (46.97)	−.17	−1.72 (374)	.087
Usefulness		2.06 (0.39)	.28	5.34 (374)	<.001
Importance		1.87 (0.36)	.28	5.18 (374)	<.001
Gender (Male=1, Female=0)		−1.29 (0.53)	−.23	−2.42 (374)	.016
Age		−0.01 (0.02)	−.02	−0.30 (374)	.766
**Education^b^**					
	Primary school	0.41 (1.78)	.07	0.23 (374)	.816
	High school	0.73 (0.75)	.13	0.98 (374)	.329
	Diploma	0.40 (0.84)	.07	0.47 (374)	.639
	Master’s degree	1.24 (0.80)	.22	1.55 (374)	.122
	Doctorate degree	3.52 (0.97)	.62	3.64 (374)	<.001
**Internet usage^c^**					
	Less than 1 hour	0.25 (1.01)	.04	0.25 (374)	.801
	3-5 hours	0.87 (0.70)	.15	1.24 (374)	.217
	More than 5 hours	1.42 (0.67)	.25	2.13 (374)	.034

^a^*R*^2^=0.28. *F*=12.29 on 12 and 374 degrees of freedom (*P*<.001).

^b^Education variables are binary variables that compare with bachelor’s degree holders.

^c^Internet usage variables are binary variables that compare with the 1- to 3-hour usage group.

**Table 4:** Types of health information sought online (n=386).

Types of health information sought online^a^	n (%)^b^
A disease or medical problem	284 (73.6)
Medical treatment or procedure	237 (61.4)
Diet, nutrition, and vitamins	219 (52.3)
Medication	202 (56.7)
Sports and exercise	196 (50.8)
A particular physician or hospital	137 (35.5)
Online support groups	35 (9.1)

^a^For each item, participants were requested to answer *yes* or *no*.

^b^Percentages represent the proportion of participants (out of the 386) who answered *yes*.

**Table 5:** Reasons for seeking health information online (n=386).

Reasons for seeking health information online^a^	n (%)^b^
To be more informed	224 (58.0)
Just out of interest	203 (47.2)
Help manage my own condition	182 (36.0)
Look for alternative or additional treatment options	146 (52.6)
Clarify information that has been given to me by a health professional	139 (31.6)
Check information discussed during a consultation with a health professional	122 (37.8)
Have information to read	107 (27.7)
Insufficient information from a health professional	79 (14.8)
Limited time with a health professional	57 (20.5)
Disagree with a health professional’s opinion	52 (13.5)

^a^For each item, participants were requested to answer *yes* or *no*.

^b^Percentages represent the proportion of participants (out of the 386) who answered *yes*.

### Social Media Channels for Health Information

Participants indicated their frequency of using various social media platforms for seeking health information online. Table 6 provides the means and SDs of participant responses, as well as the prevalence of these platforms among the general population in Kuwait. All these platforms demonstrate a relatively high level of market penetration in Kuwait led by WhatsApp and Facebook with 84% and 75% penetration, respectively [45].

Participants generally conveyed that they *Never* or *Rarely* use these social media platforms for seeking health information. The exception was YouTube, where about 50% of the participants indicated that they use the platform *Always* or *Sometimes* compared with 28% who indicated *Never* or *Rarely*. Facebook was the least desirable social media platform for seeking health information, with 71% of the respondents indicating that they never use this platform for seeking health information. This was followed by Snapchat with 45% unfavorable opinions (*Never* or *Rarely*). Twitter was also not deemed a reliable source of health information. WhatsApp and Instagram showed a relatively uniform number of responses across the 1 to 5 scale.

**Table 6:** Use frequency of social media platforms for seeking health information (n=377).

Social media platform	Mean (SD)^a^	Platform penetration in Kuwait, 2015, %^b^
YouTube	3.34 (1.31)	43
Instagram	2.96 (1.42)	43
WhatsApp	2.92 (1.53)	84
Twitter	2.72 (1.53)	41
Snapchat	2.34 (1.49)	—^c^
Facebook	1.60 (1.11)	75

^a^The mean is based on a 1 to 5 scale (1=Never, 5=Always).

^b^Source: Arab Social Media Report [45]

^c^No data.

## Discussion

### Principal Findings

To date, a limited number of studies have investigated eHealth literacy in the Middle East [17]. To our knowledge, no previous research has studied eHealth literacy rates in Kuwait, as well as the types of health information people seek and reasons why people seek health information online. The importance of eHealth literacy will continue to grow as more people gain access to the World Wide Web and as patients increasingly expect to be active consumers of health care services [16].

When the eHEALS scale was first developed in 2006, it was initially validated as a unidimensional scale [13]. This scale has since been measured and revalidated across many populations and demographics. More recent studies have shown that the 8 items reflect a 3-factor scale [43,44]. The 3 factor–scale measures *awareness* (2 items), *skill* (3 items), and *evaluation* (3 items). Nonetheless, researchers who have observed the multidimensional scale note a significant correlation among all 3 factors, which is indicative of an overarching unidimensional structure [44].

Despite some evidence that several subscales preside within eHEALS, we follow Hyde’s [43] recommendation to analyze the eHEALS as a unidimensional factor. Therefore, our analysis considers the full eHEALS, which also allows us to compare our results with that of other studies. The reported eHEALS score in this study is similar to the eHEALS scores observed in other developed countries (see Table 7). The majority of the participants indicated that the internet is a valuable source for health-related information.

Furthermore, 2 of the most important factors in predicting a participant’s eHealth literacy were the perceived *importance of accessing health information through the internet* and the *perceived usefulness of the internet* in guiding health-related decisions. This finding is consistent with previous studies and commonly associated with eHEALS [47].

The results revealed that females, on average, demonstrate higher eHEALS than males. After controlling for factors such as education, internet usage, and age, gender’s effect on eHEALS was statistically significant in our regression analysis (Table 3). In terms of using health services, previous studies observe that females visited physicians more often than males, consumed more diagnostic services, and had more hospitalizations even after excluding pregnancy-related visits [49,50]. When comparing differences in internet use, research has shown that the main drivers for internet usage among females were interpersonal communication and educational use, whereas entertainment and leisure were main drivers for males [51]. An educational motive combined with higher consumption of health services provides some logic as to why females may demonstrate higher eHealth literacy over males.

Our results indicate limited perceived value from accessing health information through social media, as demonstrated by low reliance on social media platforms (with YouTube being a slight exception). This finding corroborates previous studies, which find that *evidence related to the efficacy and effectiveness of social media is currently limited* [52]. Although the use of social media may have a promising future, there is a present need to engage and educate consumers about accessing health information via social media [53,54].

Many of the participants in this study indicated that they searched YouTube the most for health information but did not place high value on other social media platforms such as Twitter, Snapchat, and Facebook. In a classroom setting, online videos *[are] by far the most common type of social media used…with 80% of faculty reporting some form of class use of online video* [55]. This in-classroom finding may be extended to a general context as well, making online videos an effective medium for consuming information. Alternatively, it may be the ease of searchability of YouTube, which is a content-centric platform as compared with the other user-centric platforms.

**Table 7:** Similar studies examining Electronic Health Literacy Scale around the world.

Study (N)	Country	Year^a^	Electronic Health Literacy Scale	SD	Group
Britt et al [46] (422)	United States	2017	31.92^b^	5.68	College students
Sudbury-Riley et al [16] (313)	United States	2017	30.48^b^	6.40	Baby boomers
Sudbury-Riley et al [16] (407)	United Kingdom	2017	29.28^b^	6.32	Baby boomers
Sudbury-Riley et al [16] (276)	New Zealand	2017	28.72^b^	6.72	Baby boomers
Richtering et al [47] (453)	Australia	2017	27.2	4.91	Moderate-to-high cardiovascular risk
Giudice et al [48] (868)	Italy	2016	28.20	6.20	Health-literate group versus general public
Chung et al [39] (500)	Korea	2016	28.08^b^	6.43	Young adults
Tubaishat et al [17] (541)	Jordan	2016	28.96^b^	4.64	Undergraduate nursing students
Tennant et al [19] (283)	United States	2015	29.05	5.75	Baby boomers
Lee et al [15] (400)	Australia	2015	29.50	4.30	Patients with chronic health conditions
Suri et al [20] (1,062)	Singapore	2015	23.44^b^	—^c^	College students
James et al [41] (881)	Florida, United States	2014	30.40	7.80	African American adults
Choi et al [21]; Study 1 (73)	Texas, United States	2013	28.24	6.08	Low-income adults, under 60 years
Choi et al [21]; Study 2 (218)	Texas, United States	2013	25.76^b^	6.80	Low-income adults over 60 years
Mitsutake et al [18] (2,115)	Japan	2012	23.40	6.40	General population
Ghaddar et al [22] (261)	Texas, United States	2012	30.60	5.90	High school students
Van der Vaart et al [9] Study 1 (189)	Netherlands	2011	28.20	5.90	Patients with rheumatic diseases
Van der Vaart et al [9] Study 2 (88)	Netherlands	2011	27.60	5.90	Stratified sample of the Dutch population
Mitsutake et al [23] (2,970)	Japan	2009	23.50	6.50	Japanese internet users

^a^This refers to the year the study was conducted and not necessarily the year it was published.

^b^The indicated studies reported eHEALS as an average of the 8 items. To allow for a direct comparison with the other studies and our own (sum of the 8 items), we converted the average scale into a sum scale by multiplying the mean and SD by 8.

^c^Not applicable.

Facebook appears to have the least credibility as a source of health information even though it is the most widely recognized platform in Kuwait [45] and the most widely used platform for news [56]. Furthermore, an explanation for this may be a general lack of reliability of information that spreads on that platform. In fact, the spreading of fake news and rumors on Facebook has become so widespread that the company has attempted to take direct action [57]. Rumors and fake information that circulate via Twitter have also been criticized and researched in recent years [58,59]. This drives us to hypothesize that it is perhaps the perceived quality of information on a platform that drives preference for health information sources or perhaps it is the type of content; quality online videos generally take more effort to produce than news articles or blog posts and are, therefore, less likely to be authored by rumor peddlers. More research is needed to confirm or refute these observations.

In this study, most participants believed that online health information helped them make decisions about their health. This presents an opportunity for health care organizations, professionals, and government agencies providing health care services to play a more active role in monitoring, evaluating, and curating health information online [60]. It can be useful to establish policies and guidelines that ensure the credibility and quality of information similar to the HealthOnNet or DISCERN certification efforts of online health-related resources [61-63].

### Comparison With Other Electronic Health Literacy Scale Studies

In this study, we report a mean for eHEALS of 28.63 and an SD of 5.69. These findings are similar to other studies that evaluate eHEALS (refer to Table 7). Although our results are comparable with other countries, it is notable that studies conducted in some Asian countries such as Japan and Singapore report lower eHEALS scores. We acknowledge that it can be difficult to compare eHEALS because of the heterogeneity of the populations being studied. We hypothesize that the geographic location, cultural and language barriers could affect eHEALS [64] because of the lesser availability of health-related information in languages other than English. This may not be the case for the State of Kuwait as English is a secondary official language and is taught at all levels of its educational system.

### Study Limitations

Several limitations of this study need to be considered. Given the lack of previous research about eHEALS in the region, we started with the survey approach. Although the eHEALS scale is widely used to assess eHealth literacy, we recognize this as a potential limitation as the scale only captures participants' perceptions and not their actual performance [9]. In the future, more experimental studies are required to measure actual eHealth literacy performance and develop a more reliable self-assessment instrument. In addition, the results may have been influenced by the voluntary bias of the participants who were already interested in using the internet to search for information online. It may also be informative in future efforts to ask how often participants sought the health information for themselves versus for others such as a child or an elderly person.

Given our convenience sampling approach, we are not able to accurately report the survey response rates. However, this approach was useful because it provided us with a sizable sample in a reasonable amount of time. Another limitation of the study is that our sample may not accurately represent the population of Kuwait. Although our sampling approach allows for the collection of a large sample, it introduces the potential for sample selection bias. As shown in Table 1, the sample’s age distribution does not accurately reflect Kuwait’s population. Furthermore, university graduates are overrepresented in the sample. The 20- to 29-year old demographic and those with a university degree are also overrepresented in our sample, likely because 1 of the survey’s distribution channels was university colleges. Given that younger people are generally more tech-savvy and computer-literate, this sample bias may have inflated the eHEALS results. Therefore, we caution readers about the generalizability of the study and call for future research in the region to validate or update our study’s results.

### Conclusions

This study reports on eHealth literacy rates among internet users in Kuwait, as well as their perceptions about the utility and importance of the internet as a source of health information. The findings reveal high eHEALS score among the participants, suggesting that many internet users in Kuwait are confident in their ability to search for health-related information online. This high confidence and the high frequency of accessing the internet reported by the participants presents a myriad of opportunities to better engage patients digitally and conveniently. Our exploration of social media platforms as outlets for eHealth may provide guidance about how to best reach the intended audiences and stimulate further research. YouTube appears to be the most effective platform for delivering health information. Health care organizations, professionals, and government agencies providing health care services need to play a more active role in monitoring, evaluating, and curating online health information. There is a need to establish policies and guidelines that ensure the credibility and quality of information.

## Abbreviations

eHEALS: Electronic Health Literacy Scale
eHealth: electronic health
NCDs: noncommunicable diseases
SMS: short message service
